# Approaching Sarcopenic Obesity in Young and Middle-Aged Female Adults in Weight Management Settings: A Narrative Review

**DOI:** 10.3390/healthcare10102042

**Published:** 2022-10-15

**Authors:** Massimo Pellegrini, Leila Itani, Andrea P. Rossi, Dima Kreidieh, Dana El Masri, Hana Tannir, Marwan El Ghoch

**Affiliations:** 1 Department of Biomedical, Metabolic and Neural Sciences, University of Modena and Reggio Emilia, Via Giuseppe Campi, 287, 41125 Modena, Italy; 2 Department of Nutrition and Dietetics, Faculty of Health Sciences, Beirut Arab University, Riad El Solh, Beirut P.O. Box 11-5020, Lebanon; 3 Geriatric Division, Department of Medicine, Ospedale Cà Foncello ULSS2 Treviso, Piazzale Ospedale 1, 31100 Treviso, Italy; 4 Healthy Aging Center, Department of Medicine, Division of Geriatric, University of Verona, 37126 Verona, Italy

**Keywords:** clinical outcome, definition, obesity, treatment, sarcopenic obesity, screening

## Abstract

This paper presents a review of the available literature on sarcopenic obesity (SO) in young and middle-aged female adults with obesity in weight management settings. A literature review using the PubMed/Medline and Science Direct databases was conducted, and the data were summarized through a narrative approach. Firstly, some physical performance tests and questionnaires are available for screening young and middle-aged female adults with a high risk of SO. Secondly, these patients can undergo instrumental measurements such as dual-energy X-ray absorptiometry (DXA) and bioelectrical impedance analysis (BIA) to confirm or reject a diagnosis of SO, applying definitions that account for body mass. Thirdly, SO is a prevalent phenotype in females seeking weight management treatment, as well as being strongly associated (vs. non-SO) with obesity-related comorbidities that need to be promptly managed, initially with nutritional programs or/and in combination with medications. Finally, patients with SO have a reduced baseline resting energy expenditure and more sedentary behaviors, which seem to account for the relationship between SO and poorer weight management outcomes, such as a higher early dropout rate and major later difficulties in weight loss maintenance. Therefore, specific strategies for personalized weight management programs for patients with SO should be incorporated to determine a successful management of this phenotype.

## 1. Introduction

In the last few decades, there has been a growing interest in a new phenotype defined as sarcopenic obesity (SO) [1,2], which combines a reduction in muscle mass and strength with an increase in body fat deposition [3]. However, there is a shortage of studies on SO in weight management clinical settings, especially in young and middle-aged adults affected by obesity. For this reason, international scientific societies and associations addressing nutrition and obesity have recommended the consideration of SO as a clinical research priority [4].

Moreover, to date, there are still several uncertainties surrounding this phenotype. In particular, the screening tools and diagnosis criteria are still lacking for non-elderly adults [5,6]. Secondly, although a number of studies have shown a strong association between SO and obesity-related diseases, there is a lack of clarity regarding the prevalence of SO among young adult and middle-aged patients (i.e., age <60 years) with obesity in weight management settings, as well as the importance of and best approach to managing obesity-related comorbidities in this population [5]. Thirdly, the interaction between SO and weight management program outcomes is still a matter of debate. It seems to be bidirectional, with repeated failures of the latter (i.e., weight cycling) increasing the risk of SO by nearly six times [7]. On the other hand, it is still unclear whether SO (vs. non-SO) may affect obesity treatment (i.e., treatment dropout rate, weight loss and weight loss maintenance).

In the past five years, a number of well-designed research studies have been conducted in non-elderly adults with SO, especially within weight management settings. In this context, according to the data, females are more likely to seek all types of obesity treatment, including behavioral and pharmacological forms as well as bariatric surgery, and therefore they are considered to be represented to a greater extent in weight management settings [8]. In addition, SO seems to be a prevalent phenotype among females seeking weight management treatment [9].

**Figure 1:**
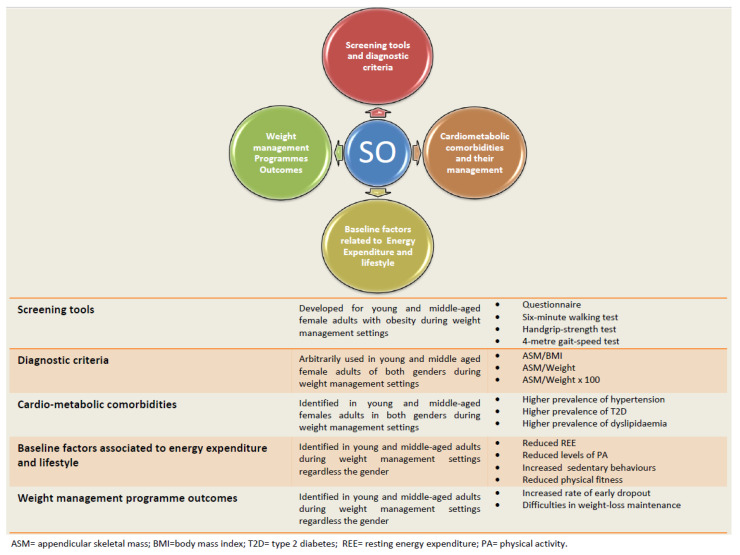
Approaching SO in young and middle-aged female adults during weight management programs.

Based on these considerations, the purpose of the current narrative review is to summarize the available literature on the topic in four main areas in this specific population, which consists of young and middle-aged females with obesity who are seeking weight management treatment, as follows: (i) to identify simple tools useful for screening individuals at a higher risk of SO, (ii) to determine an accurate assessment for confirming/rejecting a diagnosis of SO, (iii) to propose a suitable management method for SO-related comorbidities (i.e., cardiometabolic types), and (iv) to test for the relationship between SO and weight management outcomes (Figure 1).

## 2. Materials and Methods

This current review has been prepared in adherence to the narrative review guidelines [10] adopted by the Academy of Nutrition and Dietetics (Supplementary File) [11]. We searched the PubMed/Medline and Science Direct database for relevant literature using the Medical Subject Headings (MeSH) and their combinations: #1 = sarcopenic obesity, #2 = lean mass, #3 = muscle mass, #4 = muscle strength, #5 = physical performance, #6 = physical fitness, #7 = physical activity, #8 = physical exercise, #9 = energy expenditure, #10 = weight loss, #11 = weight maintenance, #12 = dropout, #13 = attrition, and #14 = clinical outcome. An additional manual search was carried out to retrieve other works that had not been identified via the initial strategy.

All analyses that evaluated SO in young and middle-aged female adults in weight management settings were considered, and included in the narrative review if they: (1) were written in English, (ii) were original publications involving research with a cross-sectional or longitudinal design, and (iii) related to prospective or retrospective observational (analytical or descriptive), experimental or quasi-experimental controlled or non-controlled studies, documenting SO specifically in young and middle-aged females with obesity within any weight management setting. On the other hand, works were excluded if they were reviews (i.e., systematic or narrative), or non-original articles (i.e., case reports, editorials, “letters to the editor” or book chapters).

There were no date limitations on the publications. However, the range of the period covered was from 2010 onward. The data were summarized using a narrative approach.

## 3. Results

### 3.1. Simple Tools for Screening Female Patients with Obesity at a Higher Risk of SO

A few questionnaires as well as physical performance tests—with suitable cut-off points—are available for screening for SO in non-elderly adults with obesity within a weight management setting. However, in a very recent pilot study composed of 23 young and middle-aged (age range 19–59 years) females, including only 13 with obesity [12], the authors developed a questionnaire for screening for SO, with items pertaining to the patients’ weight statuses and dieting practices. Every question is scored between 1 and 5 and weighted according to its importance, and a score ≥2.45 indicates a high risk of SO [12]. The researchers also found that this screening questionnaire had an acceptable sensitivity (83%) and specificity (87%) in detecting SO, defined as appendicular skeletal muscle mass/body mass index (ASM/BMI) [12] (Figure 1).

Before that, a study conducted on a sample of 147 middle-aged females affected by obesity who were seeking treatment also identified the reliability of simple tools for screening for SO in this population. These included the six-minute walk, 4-m gait-speed and handgrip-strength tests [13] (Figure 1) (Table 1):

- The six-minute walk test is usually performed to objectively evaluate functional exercise capacity and can serve as an index of patients’ daily activities. The test is performed along a straight corridor with a 30-m walking course. A brightly colored tape is used to mark the walking course every two meters, as well as the starting, end and turnaround points for each 60 m lap. Patients are instructed to walk as far as possible for six minutes and are allowed to stop or rest during the test if necessary. The participants’ respiratory rate and oxygen saturation are measured before the start and at the end of the test. The distance traveled is measured by counting the number of full laps. The cut-off point for screening for SO in this population is set at 470 m, with a sensitivity of 78% and specificity of 54% [13] (Table 1).
- The handgrip-strength test is performed to measure muscle strength using a calibrated dynamometer. The measure is usually taken on both hands and the maximum value (kg) is recorded. The cut-off point for screening for SO in this population is set at 23.5 kg, with a sensitivity of 77% and specificity of 61% [13] (Table 1).
- The 4-m gait-speed test is an objective measure of walking performance. It is usually performed on a flat course with 4 m marked out with tape. The participant is positioned with the toes just touching the start line and instructed to walk at their own pace. A stopwatch is started when the participant begins to move and stopped when the participant’s lead foot completely crosses the 4-m line. A cut-off point of 3.30 s has been identified for screening for SO in this population, with a sensitivity of 61% and specificity of 55% [13] (Table 1).

### 3.2. Accurate Assessment to Reject/Confirm a Diagnosis of SO

There has recently been a growing interest in defining SO and finding an accurate diagnostic criterion. With this aim, leading obesity societies and scientific communities, such as the European Society for Clinical Nutrition and Metabolism (ESPEN) and the European Association for the Study of Obesity (EASO), have established a new consensus for the definition of SO [14]. Although it is a valuable starting point and step forward in the direction of definition and diagnosis, it is still inconclusive, especially in regard to young and middle-aged adults [5]. In fact the majority of the cut-off points [15,16,17,18] proposed in the literature were initially developed in the elderly population with sarcopenia [16], and their use in defining SO in non-elderly adults with obesity may be highly biased, since patients with obesity tend to have a relatively high lean mass (i.e., muscle mass), and relying on parameters usually employed with elderly individuals may underestimate the prevalence of SO in a younger population with obesity [19]. Moreover, in patients with obesity, poor physical fitness is more often related to an excess of body fat and difficulty in movement, as opposed to a decrease in muscle mass [20]. This implies that the prevalence of sarcopenia may be overestimated [19].

This gap has been arbitrarily addressed, in some recent studies, when defining SO in young and middle-aged patients with obesity in weight management settings [7,9,12,13,21], relying on definitions that take body mass into consideration [19,22,23,24,25]. In other words, in addition to appendicular lean mass, these authors have employed definitions that include body weight (kg) [22,23,24,25] or BMI (kg/m^2^), and this seems to be clinically reasonable in the population in question [19,22,23,24,25,26] (Figure 1) (Table 1). However, and despite this fact, additional effort is still needed to reinforce the concept of a suitable definition of SO that will minimize false positives and false negatives. The correct identification of people with this condition using an accurate diagnostic criterion would greatly facilitate targeting people who have SO with appropriate public health measures and interventions [27].

**Table 1:** Initial screening tools and diagnosis cut-offs for SO in young and middle-aged female adults in a weight management setting.

Screening	Tool	Cut-Off for Females
Bissonnette et al. [12]	Questionnaire	≥2.45
El Ghoch et al. [13]	Six-minute walking test	470 m
El Ghoch et al. [13]	Handgrip-strength test	23.5 kg
El Ghoch et al. [13]	4-meter gait-speed test	3.30 s (gait speed = 1.2 m/s)
**Diagnosis**		
Levine and Crimmins [23]	ASM/Weight × 100	<19.43
Oh et al. [24]	ASM/Weight × 100	<23.4
Batsis et al. [26]	ASM/BMI	<0.512

SO = sarcopenic obesity; ASM = appendicular skeletal mass.

### 3.3. Management of SO-Related Comorbidities

Several investigations have reported an association between SO and obesity-related diseases, especially in young and middle-aged patients [9,21]. Two interesting examples should be mentioned. The first one, conducted in Italy in a hospitalized care setting with middle-aged patients with obesity (mean age: 45.72 ± 13.56 years, mean BMI: 37.74 ± 5.82 kg/m^2^), reported a prevalence of SO in females of 50.1%, relying on a definition that accounted for body weight [21]. The authors in this investigation found that SO was associated with metabolic syndrome and low-grade inflammation. Accordingly, they recommended a metabolic profile evaluation even in young and middle-aged patients with SO. Two years later, a study conducted in Lebanon in an outpatient weight management setting with middle-aged patients who were overweight or obese (mean age: 33.26 ± 14.65 years, mean BMI: 31.42 ± 4.94 kg/m^2^), reported a prevalence of SO of nearly 20% and a significantly higher prevalence of type 2 diabetes (T2D) and hypertension than in those without SO.

It is hypothesized that the two elements of SO (i.e., obesity combined with reduced muscle mass and strength) seem to synergistically increase the negative effects of each individual component [28]. For instance, the association between T2D and SO has recently been reported in a large systematic review and meta-analysis conducted on 60,000 individuals [29]. However, the mechanism behind the link between SO and T2D is not fully understood. Chronic inflammation, a common symptom in both conditions, obesity and sarcopenia, is known to precipitate insulin resistance [30]. Hence, the co-existence of obesity and sarcopenia in terms of the so-called “SO” phenotype may have a synergistic effect and seems to accentuate the impairment of glucose metabolism (i.e., T2D) [30]. However, the cross-sectional design employed in these works points only to a simple association between SO and some health parameters, and does not provide robust evidence for cause–effect relationships between them [31]. Therefore, these studies are not in a position to state whether SO may cause certain medical conditions, since only a few have investigated the “real” effects of SO on health outcomes using longitudinal designs. However, the fact remains that at a baseline assessment, patients with SO (vs. non-SO) have a higher prevalence of obesity-related comorbidities that need to be suitably managed. For instance, nutritional management alone (i.e., lifestyle modification programs) or in combination with specific medications—such as oral hypoglycemic, lipid-lowering and antihypertensive treatments—usually improve these conditions [32,33,34].

### 3.4. SO and Weight Management Outcomes

During any weight management program, an increase in energy expenditure (physical exercise and resting energy expenditure (REE)) and a decrease in food intake [35] are necessary for determining a caloric balance deficit to achieve significant weight loss. Moreover, in later stages, in order to maintain the weight lost over a longer period of time, a neutral caloric balance is required between the caloric intake and energy expenditure [35]. Therefore, REE and physical activity are considered important factors for the achievement of successful weight management outcomes [35] (Table 2).

Recent works have demonstrated that young and middle-aged patients with SO are characterized by impaired physical fitness (in females) [13], a more sedentary lifestyle [36] (regardless of gender), and lower REE [37] (regardless of gender) than those with obesity but not sarcopenia (SO vs. non-SO) (Figure 1). Specifically, in a study composed of 89 middle-aged patients of both genders with obesity, a lower REE was observed in patients with SO compared to those without, indicating a negative association. This was confirmed, in the same investigation, by a linear regression analysis where the presence of SO decreased REE by 1.557 Kcal/day for each kg of body weight, after adjusting for age and gender [37] (Figure 1) (Table 2).

Similarly, in a study including 111 relatively young adult males and females, those with SO were less physically active (<5000 daily steps) and walked a lower mean number of daily steps per day compared to those without SO. A multivariate linear regression analysis revealed that the presence of SO decreased the number of daily steps by 1421 after adjusting for age, gender, employment and the presence of cardiometabolic diseases [36] (Figure 1) (Table 2). Interestingly, these findings opened up new directions for research focusing on the role of baseline SO in diverting the clinical outcomes of weight management programs [38]. Namely, the main two outcomes of weight management programs during obesity treatment are:

- Early dropout: Defined as the interruption of the weight loss treatment and the main contributor to the failure of weight loss programs. The dropout rate can reach nearly 80% in some weight loss programs [39].
- Long-term weight loss maintenance (>12 months): Defined as intentional weight loss of at least 10% of body weight that is kept off for at least one year. Failure to maintain the weight lost during the weight loss phase, and a return to baseline body weight after three years of follow-up, is common regardless of the nature of the weight loss treatment and can occur in up to 70% of patients [40].

The effect of SO on early dropout as an outcome was confirmed through a six-month longitudinal study that assessed the relationship between SO and early attrition in an outpatient weight management program in young adults with obesity [41]. A significant positive association was observed between SO at baseline and early dropout. This was illustrated by a higher prevalence of SO among the “dropout group” (51.0%) vs. the “completer group” (25.8%). Further regression analysis revealed a 150% increased risk of early treatment interruption with the presence of SO at the baseline assessment (i.e., <6 months from the initiation of the program) [41] and after adjustment for gender, BMI, age at first dieting, and weight loss expectation, as well as sedentary habits (Figure 1) (Table 2).

As for long-term weight loss maintenance, another recent study assessed the relationship between SO and weight loss outcomes measured as the percentage of weight loss (WL%) at the (i) six-month and (ii) >12-month follow-up, in middle-aged adult patients with obesity [42]. Patients starting with SO at the baseline assessment demonstrated a significantly lower WL% at a >12-month follow-up, compared to those without SO (−7.34 ± 6.29% vs. −11.43 ± 4.31%; *p* = 0.024). The observed negative association between SO at baseline and WL% after more than 12 months was further confirmed by partial correlation analysis (*ρ* = −0.425, *p* = 0.009) after controlling for age, gender, and BMI. Hence, baseline SO seems to be a factor that may hinder weight loss maintenance in the long term (i.e., after more than 12 months) in an outpatient weight management setting (Figure 1) (Table 2).

**Table 2:** SO and weight management outcomes in clinical settings included in the review.

First Author	Study Design	Sample	Mean Age	Mean BMI	Treatment Setting/Follow-Up	Outcome	Finding
El Ghoch et al. [13]	Cross-sectional study	N = 147 females (54 SO/93 Non SO)	52.7 ± 12.5 years	38.3 ± 6.7 kg/m^2^	Inpatient and/or outpatient	Physical fitness	Significantly reduced in patients SO vs. Non SO.
Kreidieh et al. [36]	Cross-sectional study	N = 111 of both genders (55 SO/56 Non SO)	37.12 ± 15.58 years	36.27 ± 5.13 kg/m^2^	Outpatient	Measured daily steps	SO group displayed significantly higher prevalence of inactivity (<5000 daily steps), and they had a lower mean number of daily steps than those in the group without SO.
Tannir et al. [37]	Cross-sectional study	N = 89 of both genders (39 SO/50 Non SO)	40.62 ± 15.96 years	34.93 ± 4.68 kg/m^2^	Outpatient	REE	SO patients displayed a significantly lower REE/Weight than those in the group without SO.
Kreidieh et al. [41]	Longitudinal study	N = 103 of both genders (45 SO/58 Non SO)	35.07 ± 26.44 years	34.91 ± 6.81 kg/m^2^	Outpatient—6-month follow-up	Early dropout rate	The presence of SO at baseline increases the risk of dropout at six months.
El Masri et al. [42]	Longitudinal study	N = 46 of both genders (21 SO/26 Non SO)	44.25 ± 15.85 years	35.71 ± 4.84 kg/m^2^	Outpatient—6 and >12-month	WL% and WL% maintenance	At 6-month follow-up, patients with SO did not display a significant difference in terms of WL%, when compared to those without SO. After a longer term (i.e., >12 months), the WL% appeared to be significantly lower in the SO vs. non-SO

SO = sarcopenic obesity; REE = resting energy expenditure; WL% = weight loss percentage.

## 4. Discussion

SO is a phenotype that should be considered in a “real-world” clinical setting, particularly among young and middle-aged adults with obesity [43]. We have summarized the main findings on the topic in this review, and we now describe some important key points to consider when developing an approach to non-elderly female patients with SO.

### 4.1. Findings

Firstly, SO is a prevalent condition among non-elderly treatment-seeking female patients with obesity. Some screening tools validated specifically in this population (females) are available, i.e., physical performance tests [13] and questionnaires [12]. After the initial screening of individuals who are at a higher risk of having SO, the diagnosis can be confirmed or rejected using definitions and diagnostic criteria based on lean body mass (i.e., appendicular) measured by means of dual-energy X-ray absorptiometry (DXA) or bioelectrical impedance analysis (BIA) adjusted by body weight or BMI. The proposed “screening-diagnosis system” in our review adheres to the recently published ESPEN and EASO Consensus Statement to a large extent [14]. However, we have included tools (i.e., recently published questionnaires) [12] and cut-off points (i.e., physical performance) [13] that have been validated specifically in young and middle-aged female patients with SO in weight management settings.

Secondly, individuals with SO are at a higher risk of having obesity-related comorbidities, such as cardiovascular diseases (i.e., hypertension), T2D and dyslipidemia, that need to be suitably and promptly managed, perhaps initially through nutritional treatments (i.e., the Dietary Approaches to Stop Hypertension (DASH) diet, very low-calorie diet (VLCD), very low-calorie ketogenic diet (VLCKD), etc.), or combined with specific disease medications when necessary [44]. Thirdly, SO appears to be associated with poorer outcomes during weight management programs, in terms of higher dropout rates and major difficulties in long-term weight loss maintenance. Specific strategies should therefore be implemented to overcome these obstacles in both the early and later stages of these programs in order to improve the weight management outcomes.

### 4.2. Clinical Implications

These findings may have important clinical implications for identifying patients with SO who: (1) have a more disadvantaged baseline condition, i.e., reduced physical fitness and REE, as well as being more prone to being sedentary, (2) have a higher prevalence of cardiometabolic diseases, and (3) are more likely to drop out from treatment in the early stage and/or face more obstacles in maintaining the weight loss achieved during obesity treatment. Consequently, implementing additional strategies for this subgroup of patients is essential for obtaining better clinical outcomes.

### 4.3. Strengths and Limitations

The main strength of this narrative review is that, to the best of our knowledge, it is the first to exclusively consider a specific and homogenous population of individuals with SO, namely young and middle-aged female patients with obesity during weight management treatments. However, our findings should be also interpreted with caution due to certain limitations: firstly, the reported results were mainly derived from the same research group and therefore from one outpatient weight management unit, which implies that external validation is required from other similar settings, as well as from different ones (i.e., inpatient types) that use other modalities of treatment (i.e., pharmacotherapy, bariatric surgery). Secondly, because of the small sample sizes in these studies, these findings should be considered only preliminary and need further replication in a larger population. Finally, our review is a narrative and not a systematic or meta-analytic one, which could be considered a limitation, but only to a certain extent, since we still believe that it is premature to conduct a systematic review on SO in this specific population due to the paucity of investigations.

### 4.4. New Directions for Future Research

To this end, there is an urgent need to direct future investigations in this specific population toward: (1) the validation of additional simple tools—there are currently few—that can serve in the initial screening of individuals at a higher risk of having SO. These should be tools that are easy to use in non-specialized settings (i.e., single outpatient clinics etc.), (2) an accurate definition for SO in young and middle-aged individuals which, despite recent efforts, is not available and is still needed, (3) replicative studies to confirm the association between impaired physical fitness, reduced REE and physical activity and SO, (4) corroboration of the role of SO in determining the risk of participants dropping out of weight management programs at an early stage and (5) verification of the function of SO in hindering the maintenance of weight loss in the long term.

## 5. Conclusions

Despite the fact SO is a prevalent phenotype in young and middle-aged females with obesity, it has received little attention. For this reason, and based on our findings, the baseline screening/assessment of SO should be considered during weight loss programs in this population. Once identified, its existence should be openly discussed with patients, in the first place to highlight the disadvantages of having SO at baseline, and in the second, to propose tailored and personalized programs that may be of benefit in terms of cardiometabolic profiles as well as weight management outcomes [45].

## Data Availability

Not applicable.

## Supplementary Materials

The following supporting information can be downloaded at: https://www.mdpi.com/article/10.3390/healthcare10102042/s1. Narrative review guidelines adopted by the Academy of Nutrition and Dietetics (Supplementary File).
